# Scientific mapping of the nexus between entrepreneurial orientation and environmental sustainability: bibliometric analysis

**DOI:** 10.3389/fsoc.2024.1461840

**Published:** 2025-01-10

**Authors:** Tadesse Weyuma Bulto, Abdella Kosa Chebo, Hailu Fufa Regassa, Birhanu Chalchisa Werku, Helmut Kloos

**Affiliations:** ^1^Department of Management, Kotebe University of Education, Addis Ababa, Ethiopia; ^2^Department of Business Management, University of Johannesburg, Johannesburg, South Africa; ^3^Department of Sociology, Ambo University, Oromia, Ethiopia; ^4^Faculty of Resource Management and Economics, Wollega University, Nekemte, Ethiopia; ^5^Department of Rural Development and Agricultural Extension, Jimma University, Jimma, Ethiopia; ^6^Department of Epidemiology and Biostatistics, University of California, San Francisco, CA, United States

**Keywords:** entrepreneurial orientation, entrepreneur, bibliometric analyses, Scopus, environmental sustainability

## Abstract

Entrepreneurial orientation (EO) and environmental sustainability (ES) has recently become the subject of extensive research. The objective of this paper is to comprehensively analyze of EO and ES by conducting a bibliometric network and systematic review analysis of over ten years of publications. A total of 390 articles were identified using the Scopus and Mendeley search engines. One hundred-eighteen articles published in 53 journals between 2012 and 2021 were identified for analysis. Association analysis was conducted by author, co-author, and keyword, as well as keyword analysis by title and abstract fields, abstract field, and title field words with the highest frequency and highest relevance score under the binary counting approach. Performance, entrepreneurial orientation, relationship, entrepreneurship, entrepreneur, and business keywords were the most dominant occurrences in the abstracts. Key topics included models for entrepreneurial orientation; environmental sustainability was potentially more comprehensive in understanding the review work. This comprehensive review holds substantial theoretical significance for advancing the agenda of ecological entrepreneurial orientation and environmental sustainability. The findings of the study will help academics and researchers to identify future research directions and subject areas.

## 1 Introduction

Entrepreneurial orientation (EO) refers to a firm’s mindset and strategic activities that promote innovation, risk taking, pro-activeness, and competitive aggressiveness. Environmental sustainability (ES), on the other hand, encompasses efforts to protect the environment, conserve resources, and promote ecological balance. Societies are looking for ways to accomplish sustainable development through entrepreneurship, innovation, and environmental management has grown into a significant cause of concern (Iqbal et al., 2020). Environmental concerns have grown in importance as a component of business activities in recent years, prompting researchers and practitioners to focus more on finding viable solutions to environmental problems resulting from a range of company operations (Coelho et al., 2024). Thus, the connection between an entrepreneurial mindset and environmental sustainability is more significant than those of entrepreneurial innovation, natural resources, the green economy, and sustainable development. Because a thorough grasp of the economic landscape in the present context requires an awareness of environmental and social implications (Yadav et al., 2024). In particular, financial, environmental, and social measures were utilized to evaluate sustainability or long-term performance (Fatoki, 2019). Environmental regulation improvement also imposed several constraints on organizations engaging in globally recognized business activities and incentivized companies to implement environmentally friendly business practices (Ahmed et al., 2020). The topic of entrepreneurship research is dominated by a growing interest in sustainability (Khan and Quaddus, 2015).

Entrepreneurs who fill the gaps left by businesses and government agencies in the delivery of critical social and environmental goods and services can act as catalysts in the transition from the current economy to a sustainable economy (İyigün and Oyklu, 2015). The nature of sustainable EO is designed at the corporate level and is based on a triple bottom line sustainability model (Criado-Gomis et al., 2017). Similarly, In addition to influencing organizational behavior to match strategies with ecological objectives, green entrepreneurial orientation has become a powerful tool that helps create innovative, ecologically conscious solutions that improve corporate economic results and environmental performance (Lu et al., 2022; Yaghoubi Farani et al., 2024). This indicates the necessity of integrating EO and ES. Besides, suppliers at the top of the food chain and their sub-suppliers: the necessity of ensuring social sustainability throughout the supply chain, particularly for up-tier suppliers, cannot be overstated (Govindan et al., 2021). By putting the less fortunate on the path to meaningful lives, social entrepreneurs can help alleviate social and economic problems (Jain et al., 2019). Besides, sustainability and the preservation of the natural environment is one of the most important challenges for industrial companies in the coming years (Grobecker and Wolf, 2012). Therefore, it is essential to integrate environmental sustainability practices into EO decisions.

Today’s entrepreneurs are increasingly convinced that success cannot be attained solely by generating short-term earnings (İyigün and Oyklu, 2015). The majority of these firms are not only environmentally friendly and sustainable, but they also provide a considerable number of essential ecosystem services (Shahidullah and Emdad Haque, 2014). However, most studies in business disciplines focus solely on studying entrepreneurship, while studies in environmental sciences solely focus on studying environmental protection without considering business issues. It is necessary to consider both concepts together in order to provide a comprehensive understanding of both profit-making and environmental protection. According to Hörisch (2015), sustainability and the protection of the natural environment are key challenges that industrial companies will encounter in the future. As a result, it is crucial to incorporate environmental sustainability practices into EO decisions.

Furthermore, the public debate on corporations’ environmental orientation has risen, and there is a lack of understanding of the repercussions of this orientation, particularly in terms of its impact on company network behavior (Dickel et al., 2018). On the other hand, small businesses’ environmental sustainability orientation can be explained by their EO, according to the company’s natural resource-based perspective (Roxas et al., 2015). However, small and medium-sized businesses alone cannot fulfill environmental sustainability targets (Ben Amara and Chen, 2020).

Previous scholars may not have fully addressed the growing focus on green entrepreneurship, which takes into account both corporate profitability and environmental concerns. Despite the growing research interest in entrepreneurial orientation (EO) and environmental sustainability (ES), there is a lack of comprehensive studies that link EO to environmental sustainability in developing countries (Lu et al., 2022). The aim is to strike a balance between corporate growth and environmental sustainability (Meirun et al., 2020). Because corporate activities have been regarded as a crucial driver of economic, social, and environmental sustainability, researchers have accepted the link between business size and the environment (Golsefid-Alavi et al., 2021). Moreover, the key goal of environmental entrepreneurs is expected to deal critically with rising institutional, customer, and environmental constraints in order to achieve environmental sustainability (Makhloufi et al., 2022) and to be incorporated in the entrepreneurial programs of firms. Similarly, it is vital to consider environmental issues in the context of businesses (Yaghoubi Farani et al., 2024).

This study can assist both practitioners and policymakers in various ways. First, it explores performance analysis and scientific mapping of EO and ES. A literature search using well-known databases such as Scopus was conducted to identify research topics related to performance, EO, entrepreneurship, entrepreneurship, business, and sustainable development. Based on this, this study establishes a baseline of data on the integrated topic for future comparisons and for policymakers to incorporate environmental issues into their entrepreneurial decisions. The study also covers every facet of EO and ES, rather than focusing on just one set of variables, and recommends aspects that could be helpful for managerial decision making. Therefore, this study contributes to practitioners and future researchers by consolidating and offering a comprehensive understanding of the subject, which has been fragmented and debated previously.

Second, despite the importance of integrating EO and ES, there is a lack of research on how these concepts can be connected effectively. Although previous research has shown some links between EO and environmental factors and entrepreneurial intention, these studies have frequently either looked at these concepts separately or have only focused on specific outcomes like intention. Research that methodically examines the ways in which integrating EO and ES as related concepts affects more general, associated themes including sustainability performance, green innovation, and strategic entrepreneurial practices is lacking. By performing a thorough bibliometric analysis and systematic review, this study fills this gap by providing a unique map of the scientific advancements in EO and ES over the previous ten years. Additionally, existing studies have failed to clearly establish how the combined ideas of EO and ES relate to other relevant themes and concepts. These gaps highlight the need for further research and integration of EO and ES in order to address the complex challenges posed by various factors. Third, this study is among the first to provide significant data on the most influential sources, authors, institutions, and countries in the field. It also identifies key ideas related to EO and ES as well as trends in publications and thematic evolution.

Considering the above-mentioned points, the purpose of this study is to provide a more quantitative application of bibliometric techniques that will provide author and keyword analysis through the examination of networks of co-authorship and co-occurrence terms involved in the production of research results. Thus, the objective of this study is to examine the existing research productivity and scientific mapping of co-authorship and word co-occurrences in EO and ES subjects and to show future research trends. While EO can drive ES initiatives, it may also lead to challenges, such as balancing short-term profitability with long-term sustainability goals. Firms must navigate these trade-offs carefully. In line with this objective, the study answers the following research questions:

1) What are the most productive journals and authors?
2) What are the major topics studied in EO and ES subjects?
3) What are the key topics to be studied in future research?

### 1.1 Theoretical research foundation

Marx argued that capitalism’s root causes include resource loss and a disconnect from the world. He proposed a more compassionate, ecologically conscious social structure, advocating for a balanced distribution of people and a healthy link between industrial and agricultural production for a sustainable society (Hannigan, 2023). Allan Schnaiberg’s treadmill of production theory suggests that modern industrial societies prioritize economic growth, resource extraction, manufacturing, and consumption, leading to environmental degradation, but is criticized for its challenges in promoting sustainable development within the capitalist system (Pigman and Higgins, 2015). Moreover, ecological modernization theory suggests that integrating business practices and technological innovations can lead to economic growth and environmental sustainability. It advocates for market-based solutions, government intervention, environmental activism, and entrepreneurship, promoting long-term sustainability (Hannigan, 2023). The objective of ecological modernization is to reduce the negative environmental effects of future production processes by employing cutting-edge technologies to ameliorate environmental degradation within institutions (Auriffeille, 2020).

Given the importance of integrating environmental aspects into business decisions, ecological concerns should be incorporated into the corporate goals of organizations (Lu et al., 2022). The heightened awareness in sustainable development coupled with globalization has created immense aspiration, enthusiasm and interest in the trajectory of sustainable entrepreneurship (Hooi et al., 2016). For instance, China, the world’s largest energy consumer, has given sustainable development unprecedented emphasis in its 12th five-year plan (Lei et al., 2019). They consider the Manufacturing Sustainability Disclosure Index as a tool for micro, small, and medium-sized businesses to meet their social and environmental obligations (Singh and Roy, 2019). Furthermore, environmental intention is a key predictor of environmental behavior, but there is little theoretical and empirical evidence on it, especially in developing countries (Tounés et al., 2020). Environmental challenges are increasingly becoming a component of corporate performance, and policymakers and executives have begun to appreciate the value of green innovation in achieving long-term success (Smes, 2021). When compared to green purchasing and reverse logistics, green collaboration with suppliers had the largest influence on operational performance (Mafini, 2017).

As the number of environmental issues created by business activities grows, entrepreneurs are under more pressure to employ environmentally friendly measures in their businesses (Handrito et al., 2021). The results of this study are not an artifact of environmental variation because business groupings are resilient across environments (Atuahene-Gima and Ko, 2014). Entrepreneurship is linked to a dedication to long-term growth, notably in terms of the environment, human resources, and community involvement (Ayuso, 2017). Circular entrepreneurship is defined as the process of identifying and exploiting possibilities in the circular economy (Cullen and De Angelis, 2021). In the age of Industry 4.0, environmental and social implications are critical, particularly for innovative technology (Khofiyah et al., 2021). Moreover, the rise in environmental regulation also enforced several limitations on organizations to follow the globally accepted business activities and incentivize firms for implementing eco-friendly business methods (Ahmed et al., 2020). The entrepreneurial orientation of companies (measured by their innovativeness, pro-activeness, risk-taking, autonomy and competitive aggressiveness) and sustainable development are not mutually exclusive (Gaweł, 2012).

Sustainability-related entrepreneurship has become a significant aspect of entrepreneurship in order to operate greener and more sustainably (Gast et al., 2017). An examination of moderation effects reveals that regulatory, normative, and cognitive factors have positive moderation effects on the relationship between opportunity-based entrepreneurship and sustainable development environmental quality (He et al., 2020). Tourism organizations’ cooperation and creativity, employee culture, technology infrastructure, tourism intermediary sustainability practices, and top management support all have a substantial impact on the adaptation of sustainable practices (Islam and Zhang, 2019). The younger generation’s interest toward green business had a key positive impact on sustainability orientation and education (Soomro et al., 2020). The concept of going green was progressively adopted by the public (Ye et al., 2020). There are other principal motives for firms, environmental innovation mediates the positive effect of environmental entrepreneurial orientation on firm performance; stakeholder pressure positively moderates the influence of environmental entrepreneurial orientation on environmental innovation, and environmental entrepreneurial orientation influences firm performance through environmental innovation (Guo and Wang, 2022). However, there can also be challenges in integrating the entrepreneurial orientation and environmental sustainability. Potential issues include budget constraints, policy complexity, and the need for cultural shifting within organizations. Furthermore, aligning the entrepreneurial orientation with environmental sustainability practices can lead to the positive outcomes such as reduced resource consumption, improved brand reputations and enhanced the stakeholder relationships.

## 2 Methodology

### 2.1 Study setting

The purpose of this study is to investigate the thematic integration of EO and ES. To achieve this, we employ a systematic literature review approach. This method is chosen because it follows a structured process that can be transparently disclosed and replicated, reducing review ambiguity and bias (Kumar et al., 2023). Systematic literature reviews are increasingly used to synthesize existing literature in a field (Kraus et al., 2020). Additionally, systematic literature reviews, bibliometric methods allow for the measurement of scientific activity using quantitative and objective methods (Glinyanova et al., 2021). Moreover, bibliometric techniques benefit from the use of quantitative and statistical measures and technology, making them more comprehensive and less subjective than other literature review variants conducted manually (Mukherjee et al., 2022). Thus, bibliometric analysis has become a popular and rigorous method for exploring and analyzing large volumes of scientific data. It helps us uncover the evolutionary nuances of a specific field and identify emerging areas of research. However, its application in business research is relatively new and, in many cases, underdeveloped (Donthu et al., 2021).

### 2.2 Data searching strategies and gathering

**Figure 1:**
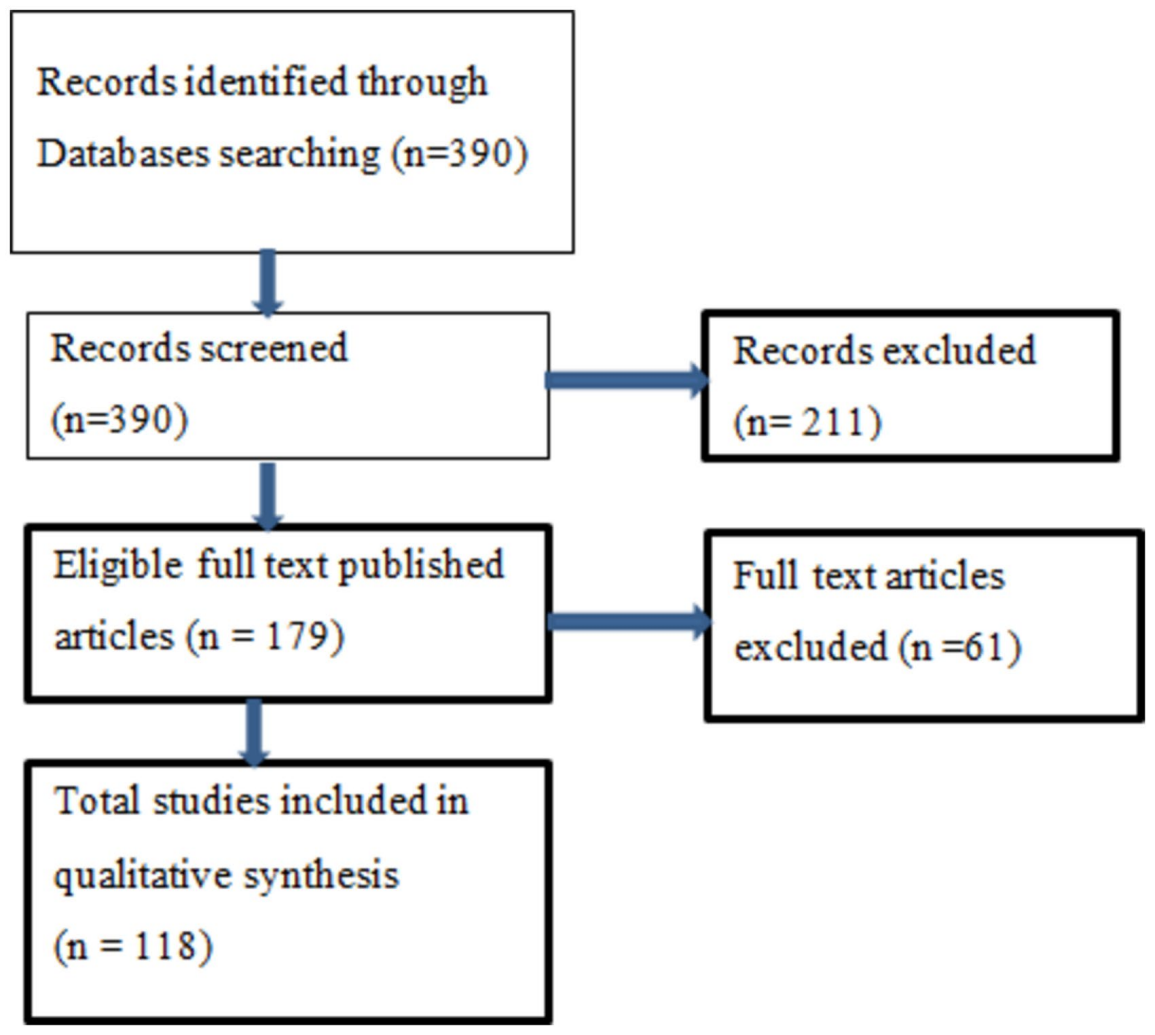
The flow chart of the study selection process.

The data was gathered using the Scopus and Mendeley search engines, with the following search terms: nexus between entrepreneurial orientation and environmental sustainability covering the period 2012 to 2021. The Scopus data base is an important database for accessing global academic information. The literature reviews on entrepreneurial orientation and environmental sustainability were combed through. The Preferred Reporting Items for Systematic Reviews and Meta-Analyses (PRISMA) guidelines and charts are used to create these search variables (Andrade-valbuena et al., 2018; Liberati et al., 2009; Pahlevan-Sharif et al., 2019). Word terms from the title field, keywords, title and abstract fields, and abstracts field were used as search phrases. The Mendeley search engine and the Scopus search engine yielded a total of 390 articles. The bibliometric study was conducted on 118 published journal articles. The research’s chronology was created based on the review’s criteria and the three main elements of the systematic review in Figure 1.

### 2.3 Inclusion and exclusion criteria

Data for this review were gathered using the Scopus and Mendeley search engines, and only English-language journal articles were reviewed and exported in RIF formats. All publications published between 2012 and 2021 were chosen for the investigation because the goal of this research was to better understand the concepts of corporate governance and environmental sustainability. For two reasons, 2012 was chosen as the starting year for the sample of goods. First, prior to 2012, the publications found in our study were few and had similar themes. Beginning in 2012, selected periodicals published articles with a central focus on entrepreneurship and environmental sustainability. Second, using the search phrases entrepreneurship and environmental sustainability, articles were found using the Mendeley search engine of each of the selected publications, whereas search on Scopus from title, abstract and keywords based on “entrepreneurship orientation” AND “environmental sustainability” OR “entrepreneurship orientation” AND “sustainability” OR “sustainable development” AND “entrepreneurial orientation” OR “sustainable entrepreneurship AND “entrepreneurship” OR “entrepreneurship” AND “Environmental Entrepreneurship.” In the first phase, we used co-occurrence analysis techniques to perform a quantitative bibliometric analysis of the 390 peer-reviewed articles. Subsequently, 329 journal papers were chosen, and only 118 journal articles were assessed in the second phase for inclusion or exclusion; all scientific journal articles published on entrepreneurial orientation and ecological sustainability were included; non-English text research and all documents published on non-journal articles were excluded. Following this procedure, 242 items were eliminated and 118 were chosen for bibliometric analysis.

### 2.4 Data cleaning and standardization

In systemic reviews, data consistency and purification were critical. The standardization of the codes (authors, co-occurrence, and keywords) was done manually, and each article’s information was examined individually (Lojo et al., 2018). The list of articles from mendeley searching engine was first transferred using Mendeley Reference/Citation Manager Software 1.19.8. The software interface was used to standardize the authors’ first and last names (single authorship). Second, because the article’s keywords needed to be consolidated, related terms were reduced and standardized. The RIF’s data sources were retrieved using the abstract field, ignoring the structured abstract label and copyright assertion.

### 2.5 Data analysis

Mukherjee et al. (2022) propose that performance analysis and science mapping results can serve as a starting point and complement other review techniques in advancing theory and practice. In order to do so, VOSviewer version 1.6.17 was used to plot the created maps, network visualization, bibliography, and (link) (Van Eck and Waltman, 2021). Different clusters are used to organize the items. In such steps, the library was exported as a plain text data file, and the data was processed for sophisticated bibliometric analysis using Mendeley software version 1.19.8.

## 3 Results

**Figure 2:**
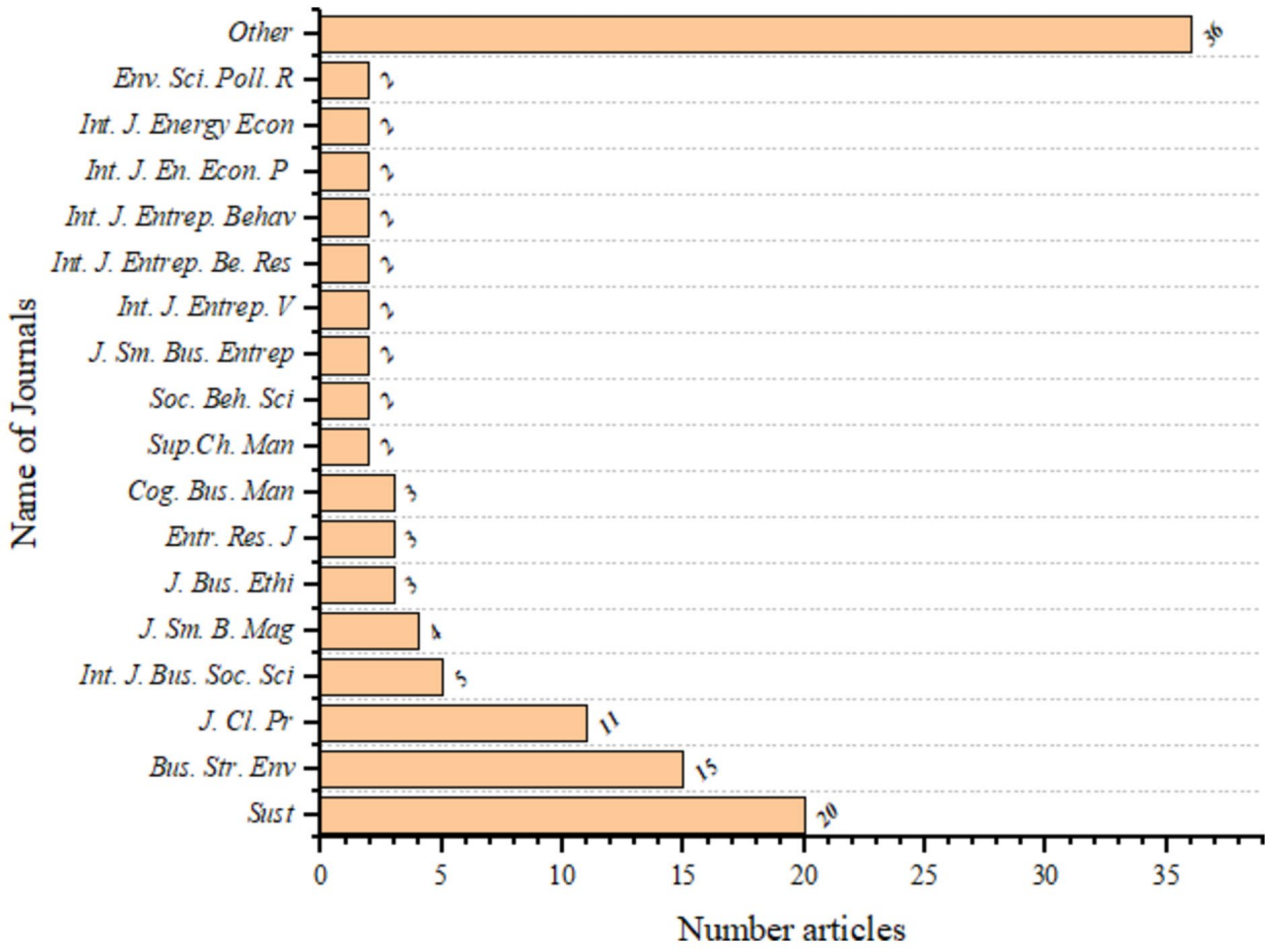
Overview of the most popular journals based on the number of scientific publications on corporate orientation and environmental sustainability. See the full names of the journals given in (Appendix 1).

This analysis examines the evidence for entrepreneurship and its contribution to environmental sustainability by synthesizing findings from 118 studies that meet the eligibility criteria. The top three peer-reviewed journals are Sustainability (20 publications), Journal of Business Strategy and Environment (15 publications), Journal of Cleaner Production (11 publications), and International Journal of Business and Social Science (5 publications), as shown in Figure 2. This indicates that the subject of integrating EO and ES is a multidisciplinary study published by a variety of journals from different disciplines. Besides, the issue is a top issue and a concern for top scholars since the main journals publishing the study are top journals with high impact.

**Figure 3:**
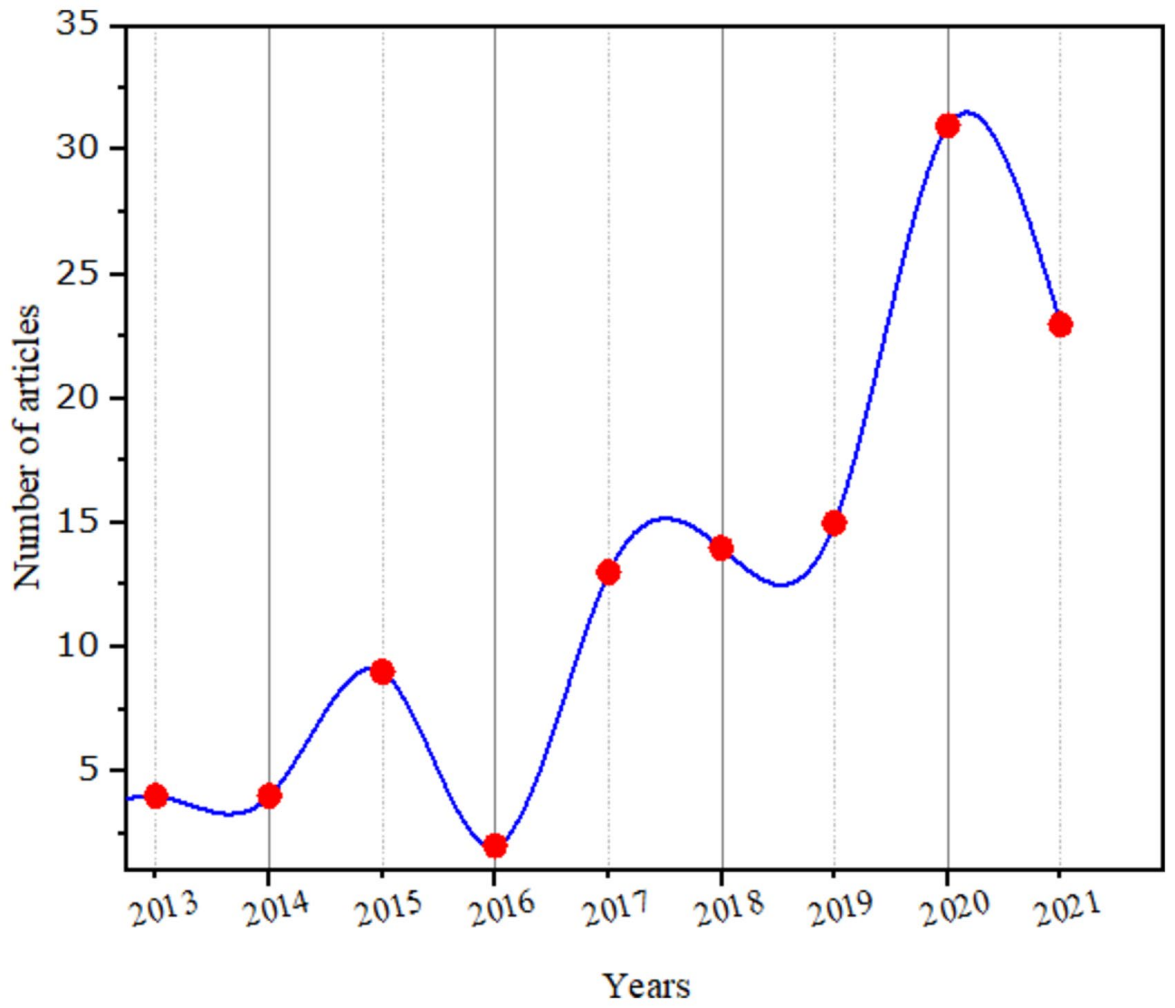
Annual publications trends during study period.

A total of 118 records were retrieved in this study, excluding non-journal articles. Based on annual scientific production the published articles were increased from 2012 and 2021 on the relationship between Entrepreneurial Orientation and Environmental Sustainability. The publications have gradually increased, with a flow starting in 2016 and 2021 on Entrepreneurial Orientation and Environmental Sustainability as well as the distribution of articles published per year in Figure 3.

**Figure 4:**
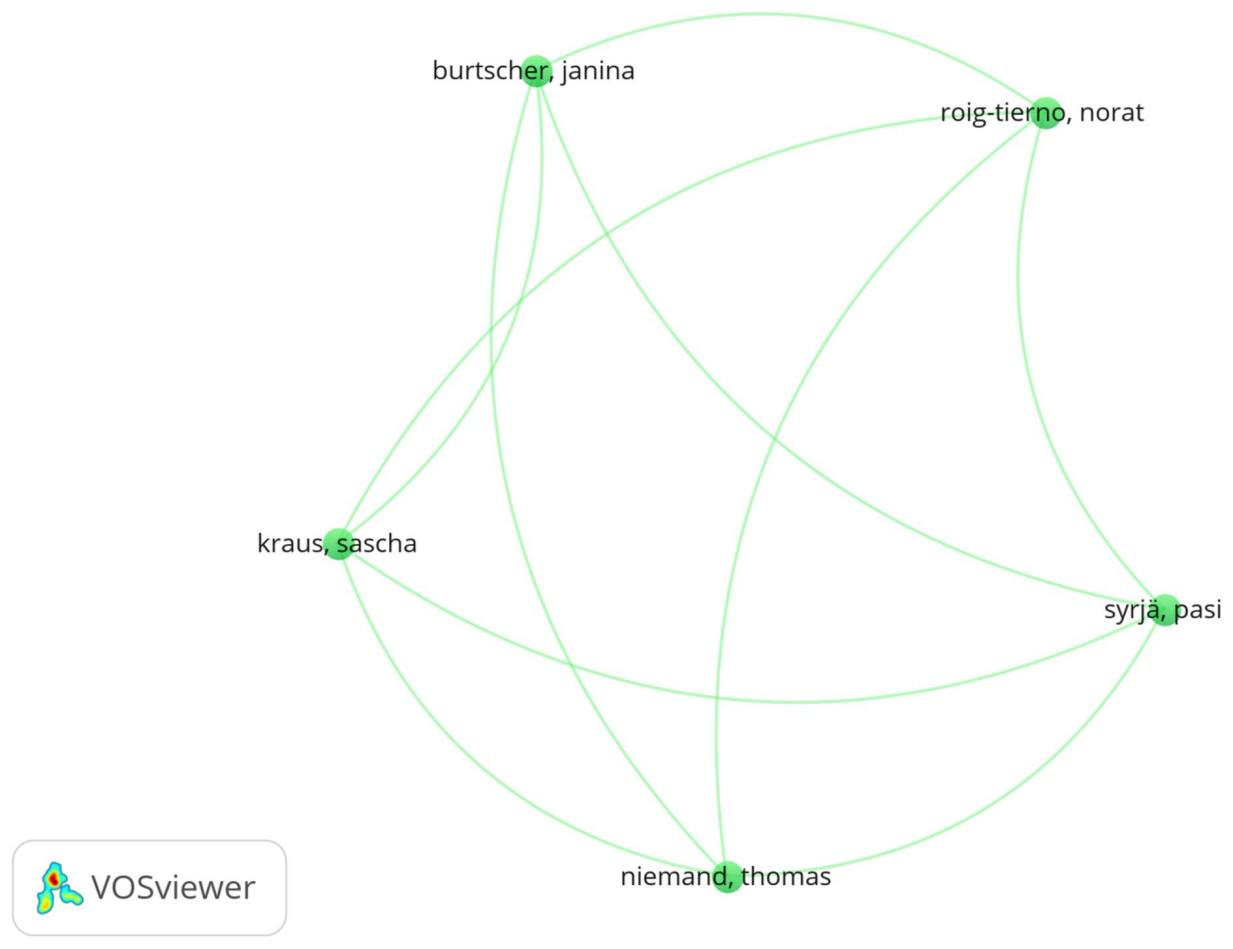
Network of co-authorships with author/s. Where: the minimum number of occurrences of terms 2; of the 28 terms, 33 met the thresholds and 25 terms were retained. The distance between two journals in the visualization roughly indicates the relationship of the journals in terms of co-publication links.

As illustrated in Figure 4, the number of published articles slightly increased substantially from 2012 to 2021 on the entrepreneurial orientation and research collaboration of environmental sustainability.

**Figure 5:**
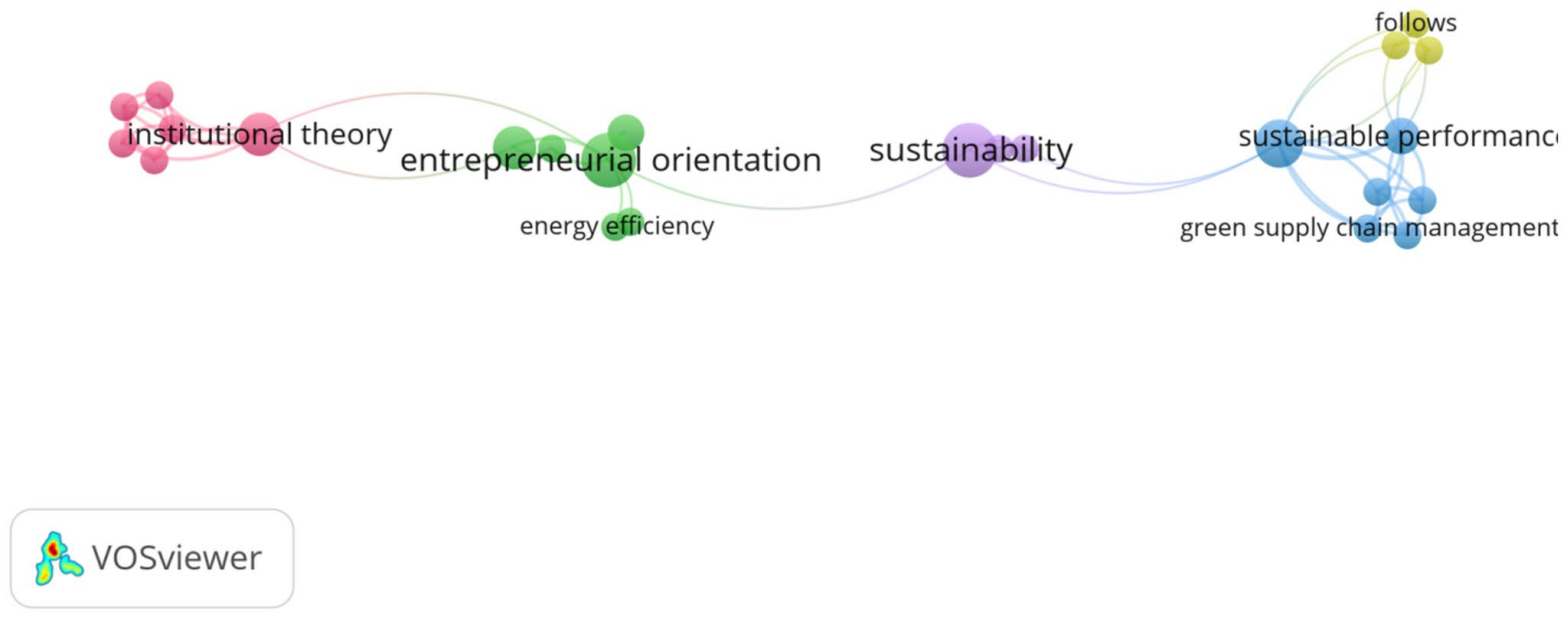
A co-occurrence network of the most frequently used keywords (full counting analysis). Where: of the 183 terms, 25 were preserved because they fit the criteria. Terms must appear a least of twice; the distance between two journals in the graphic essentially represents how linked the journals are based on relationships between co-citations.

Figure 5 shows the findings of the co-authorships with author analysis. The original dataset consisted of 118 published articles. The visualized bibliometric network represents five interconnected items in a cluster with unique labels based on co-authorships from the original dataset of 280 authors, which can be reduced to a group of five author clusters by selecting two in the one author minimum number of documents and meeting 33 thresholds, while 33 author numbers were obtained in covered subjects. However, documents with a significant number of authors or a maximum of 25 author numbers per document are ignored (Appendix 2). The most popular authors are displayed in a larger font, while the most connected authors are displayed in a smaller font. Burtscher Janina, Kraus Sascha, Nobody Thomas, Roig-tiermo Norat, and Syrja had the most tightly networked co-authorships with authors on entrepreneurial orientation and ecological sustainability.

The stronger the association between two journals is, in general, the closer they are to each other. Lines also show the strongest co-citation relationships between journals. The correlation between terms in the article sample is visualized using co-word analysis. The minimum number of times a keyword appears. Two (2) of the 183 keywords meet the standards, and 25 keyword counts were collected in addressed themes, resulting in a set of five clusters with 25 items. The most popular keywords are larger, while the themes that are most closely connected are grouped in the middle of Figure 5. Green entrepreneurial orientation (16 links with other keywords), sustainable performance (14 links), and institutional theory (12 links) are the most often examined components, with the largest occurrences. Entrepreneurial orientation (6), sustainability (6), and green entrepreneurial orientation (6) were among the buzzwords (5) (Appendix 2).

**Figure 6:**
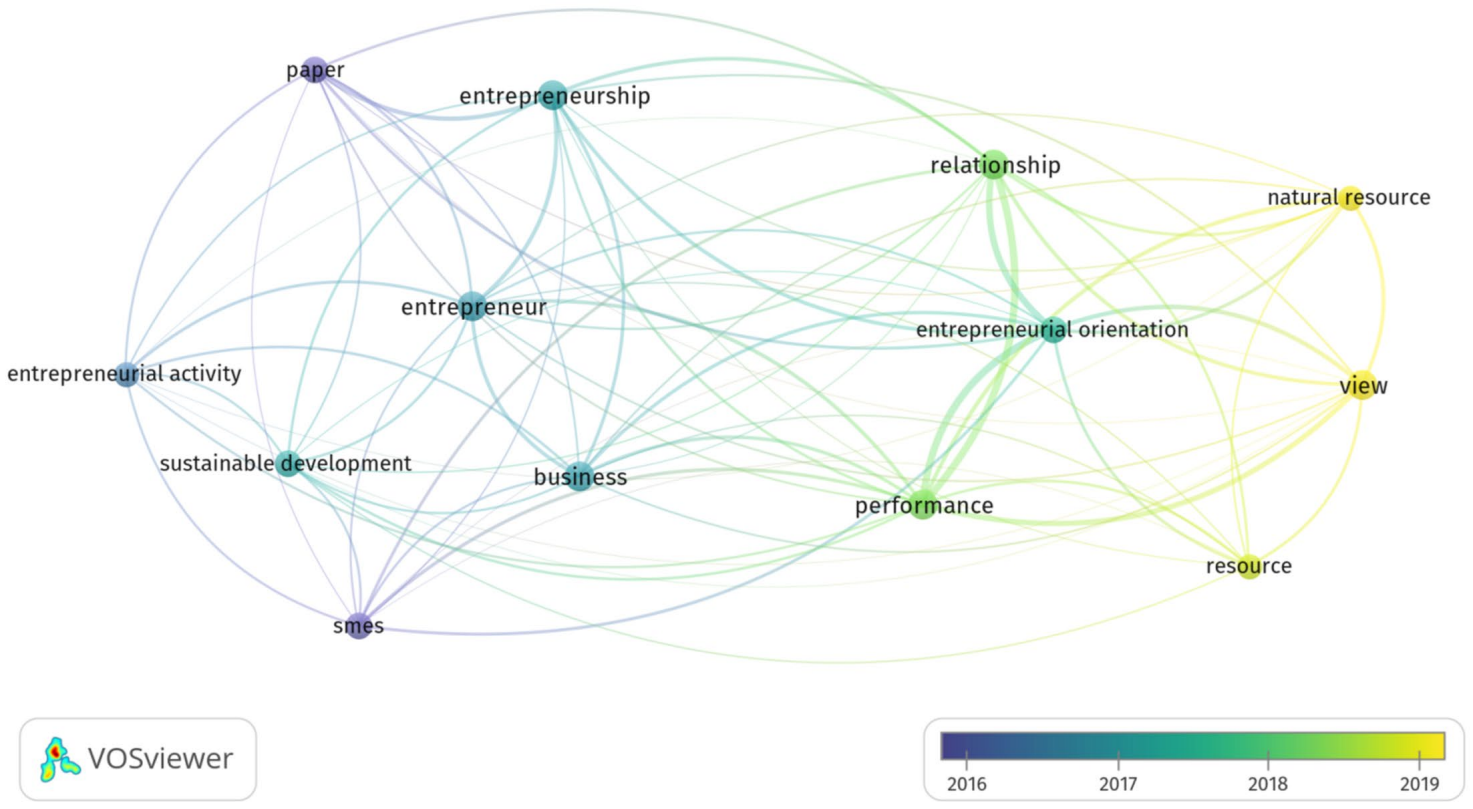
Overlay visualization occurrences of terms in abstract fields based searching of the total number of articles. The minimum number of occurrences of terms 10; of the 1,538 terms, 22 meet the thresholds, and 60% are the most relevant terms and 13 numbers of terms were obtained.

As shown Figure 6, the main overlay visualization occurrences of terms by the abstract field showing the occurrences and relevance in the field of entrepreneurial orientation and environmental sustainability by binary counting. The top six in terms of total occurrences in abstract field were performance (33), entrepreneurial orientation (32), relationship (29), entrepreneurship (26), entrepreneur (19), and business (18). However, the highest relevance scores were entrepreneurial activity (2.35) and sustainable development (1.24) among all abstract fields terms, respectively (Appendix 3) (see Figure 6).

**Figure 7:**
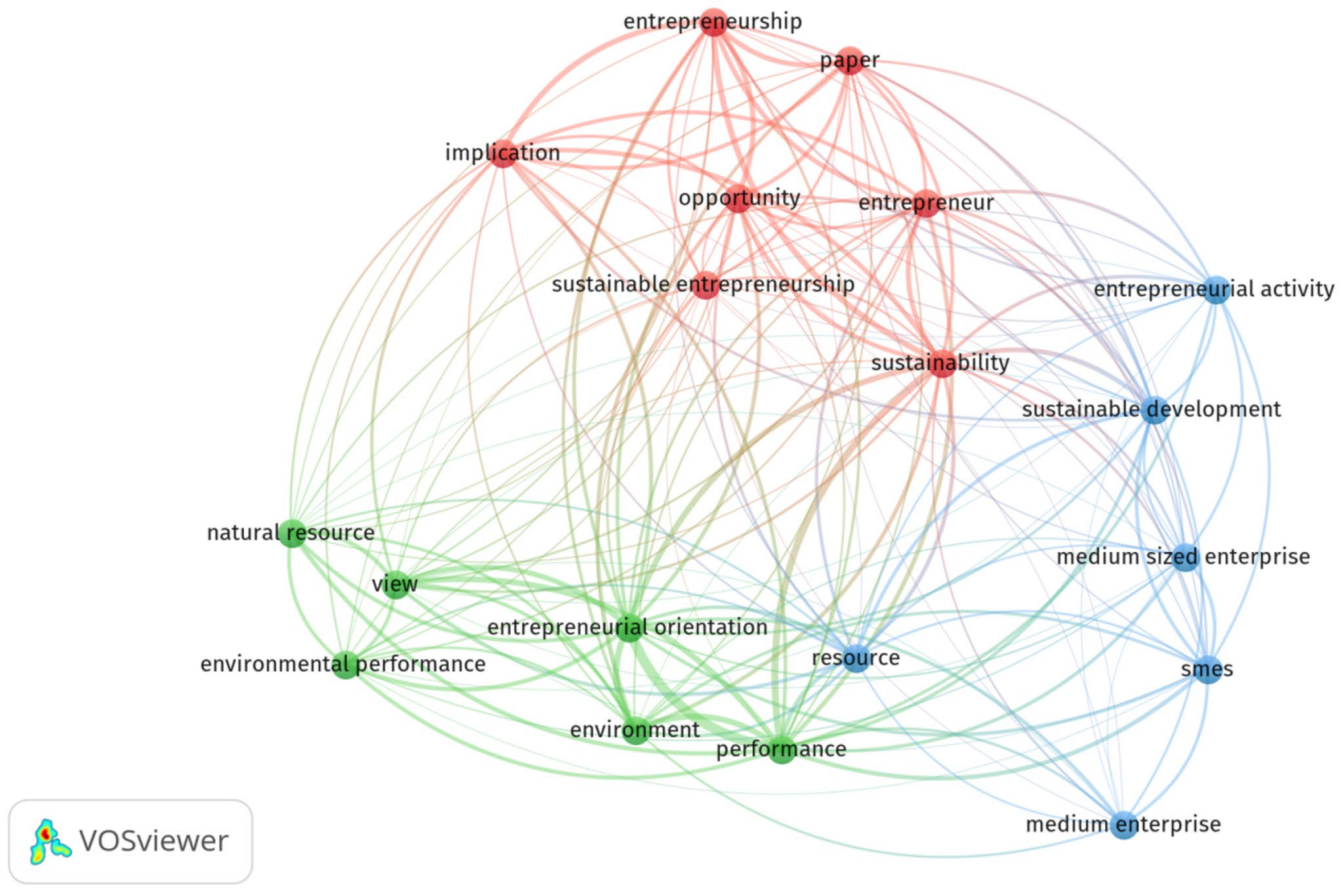
Network visualization occurrences of terms in title and abstract fields based on the search of the total number of articles. The minimum number of occurrences of terms 10; of the 1719 terms, 31 meet the thresholds and 60% are the most relevant terms and 19 terms were identified.

Figure 7, demonstrates the main network visualization occurrences of terms used by the title and abstract fields shows the occurrences and relevance in the field of entrepreneurial orientation and environmental sustainability by binary counting. The top six in terms of total occurrences in title and abstract field were entrepreneurial orientation (47), performance (42), sustainability (40), entrepreneurship (27), environment (21), and implication (21). However, the highest relevance scores were natural resource (1.67), entrepreneurial activity (1.54) and medium enterprise (1.50) among all terms of the title and abstract field, respectively (Appendix 4).

**Figure 8:**
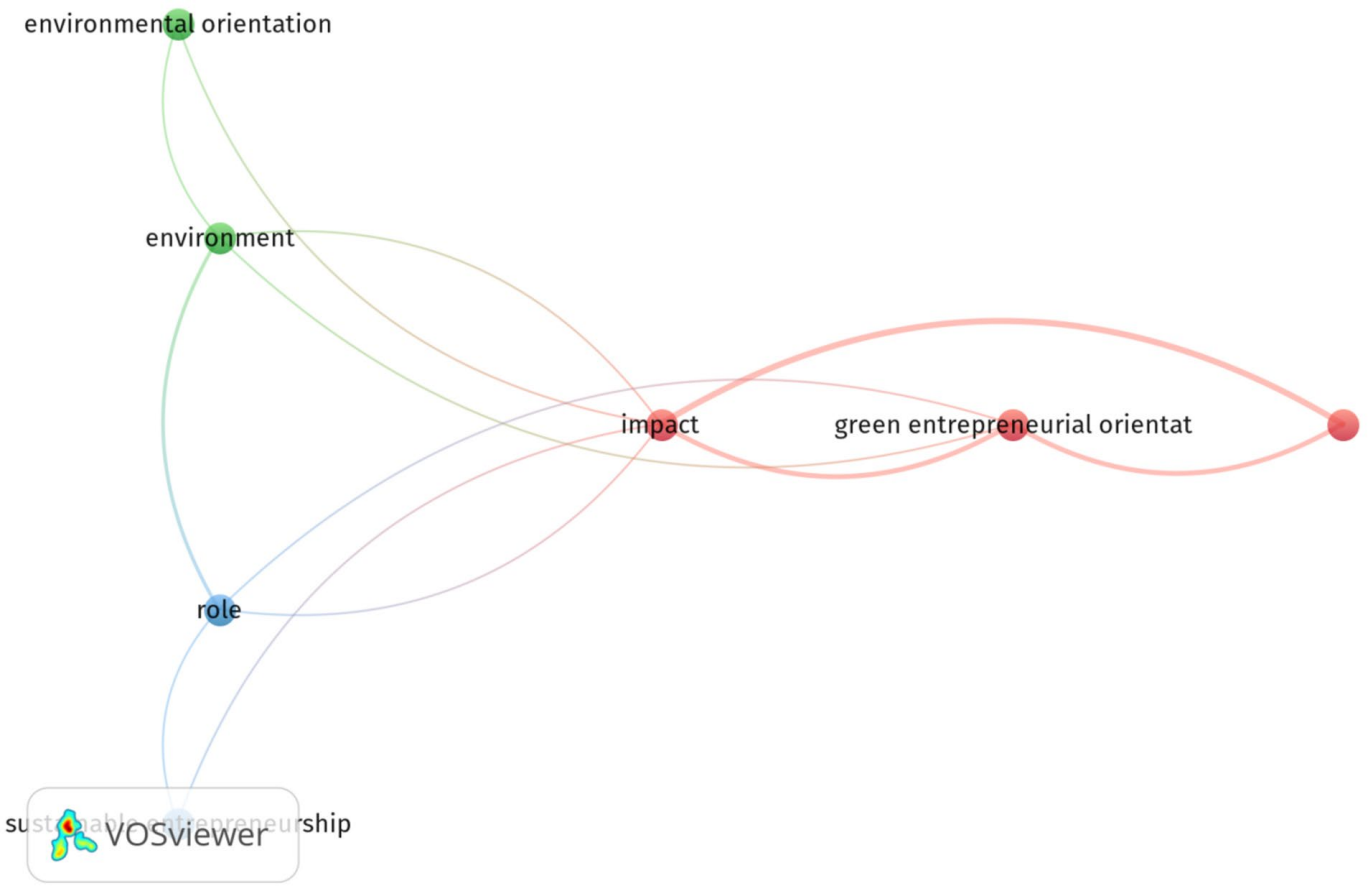
Network visualization occurrences of terms in title field based searching of the total number of articles. The minimum number of occurrences of terms 5; of the 358 terms, 18 meet the thresholds and 60% are the most relevant terms and 11 terms were preserved.

As indicated in Figure 8, the major network visualization occurrences of terms used by the title field show the occurrences and relevance in the field of entrepreneurial orientation and environmental sustainability by binary counting. The top five in terms of total occurrences in the abstract field were role (14), impact (12), SMEs (9), sustainable development (9), and green entrepreneurial orientation (7). However, the highest relevance scores were for SMEs (2.47) and sustainable development (2.47) among the terms of title field.

## 4 Discussions

The relationship between entrepreneurial orientation (EO) and environmental sustainability (ES) is a complex and multifaceted topic that has garnered significant attention in recent years. EO, often characterized by risk-taking, innovation, pro-activeness, competitive aggressiveness, and autonomy, is a key driver of economic growth and development. ES, on the other hand, is concerned with preserving the natural environment for future generations. In all disciplines of scientific and technical studies, there is strong evidence that collaboration has become the norm (Bozeman et al., 2013). Inter-company collaboration positively effects collaboration on corporate social responsibility spending, according to a study conducted in Ghana, and this relationship is increased when corporate orientation is larger in volatile settings (Adomako, 2020). Furthermore, the significant acknowledgment of university-industry relationships as promoters of economic advancement, innovation, and competitiveness fostered a continued commitment to research (Skute et al., 2017). Other finding support that (Bouguerra et al., 2022), entrepreneurial orientation, strategy, and environmental sustainability by providing logic rooted in stakeholder theory of the conditions under which multinational companies’ entrepreneurial orientation in emerging markets prioritizes and privileges environmental collaboration with suppliers. As study conducted in Malaysia, students have a moderate level of sustainable orientation while entrepreneurship is at a high level (Nordin et al., 2018). The development of global metrics for evaluating university research performance has been accompanied by increasing attention to key performance metrics for individual disciplines (Pojani et al., 2022).

According to the study, sustainable entrepreneurship accounts for 58.6 percent of the variation in international family company success, while the environment plays a positive moderating influence, accounting for 73.5 percent of the variance (Perlines et al., 2018). Green product innovation is a cornerstone of environmental management (Andersén, 2022); (Soo Sung and Park, 2018). Sustainability and entrepreneurship are often regarded as binary concepts that have a tradeoff relationship, meaning that the higher the social and environmental consideration, the lower the private and economic benefits. The study revealed that a high level of stakeholder integration strengthens the indirect association between entrepreneurial direction and new business performance (Amankwah‐Amoah et al., 2019). The interplay between entrepreneurship orientation (EO) and alliance orientation (AO), based on the Dynamic Capability perspective, promotes Dynamic Capability and hence the long-term international performance of SMEs (Street et al., 2021).

Furthermore, their entrepreneurial strategy alignment allows them to take a more proactive approach to environmental sustainability measures, which leads to improved commercial performance (Roxas et al., 2015). By putting the less fortunate on the path to meaningful lives, social entrepreneurs can help alleviate social and economic problems (Jain et al., 2019). SEO is tackled through an organizational paradigm of strategic orientations, bound by competitive culture and multiple orientation viewpoints, according to the Dynamic Capabilities view (Criado-Gomis et al., 2017).

Green supply chain management strategies have a major beneficial impact on sustainable business performance when led by a green entrepreneur with a market focus. Some of the major elements influencing financial, social, and environmental success include identifying innovation and entrepreneurship, government policies, and lean manufacturing methods (Beatriz et al., 2019).

The most significant aspect for innovation orientation, customer orientation, supplier orientation, and network orientation is economic and ecological sustainability performance (Nawi et al., 2020). Sustainable Entrepreneurial Orientation (SEO), which can play a crucial role in aligning the organization towards sustainable development and achieving sustainable business performance, can explain the association between manager age and sustainable performance (Ameer and Khan, 2020). As study conducted by Nigerian students (Ibrahim and Lucky, 2014), entrepreneurial orientation, entrepreneurial skill and environmental factor and their connection had relationship with entrepreneurial intention. Characteristics of entrepreneurs who enjoyed sustainable success in operating small businesses could be categorized under six dimensions: a business spirit, pro-activeness, competitive advantage, sustainability, human capital and firm performance (Amornpinyo, 2018).

Entrepreneurship research has widely explored various aspects of family businesses (Srivastava et al., 2024). EO has a significant positive effect on disruptive innovation and that deployment of a digitalization strategy is perceived as a metaphorical cage for disruptive innovation among highly entrepreneurially oriented firms (Kraus et al., 2023). Government support policies both financial and nonfinancial can directly impact SME performance or indirectly by developing an entrepreneurial orientation (Prasannath et al., 2024). Moreover, sustainable leadership and sustainable entrepreneurship are interrelated concepts that play a key role in promoting responsible business practices and formulating solutions to contemporary socio-environmental challenges (Ribeiro and Leitão, 2024). From a social perspective, embracing sustainable practices could positively impact, employment, environmental responsibility, economic stability, and consumer perception (Tolossa et al., 2024). Sustainable entrepreneurship has grown in both academic and business circles, with the aim of promoting the creation of environmentally and socially responsible enterprises (Valencia-Arias et al., 2024).

## 5 Conclusion

The relationship between Entrepreneurial Orientation (EO) and Environmental Sustainability (ES) is increasingly important in today’s business landscape. There is much proof that environmental sustainability and entrepreneurship go hand in hand. Based on the research, the authors were able to connect the diverse perspectives of entrepreneurial orientation with studies on collaborative environmental sustainability. This study examines the relationship between EO and ES, which is crucial for the development of a green economy and long-term growth. Although the existing literature has highlighted the strong link between these two, it is not yet fully understood. It is worth noting that the most frequently occurring keywords in the abstract are performance, entrepreneurial orientation, entrepreneurship, entrepreneurship, and business. However, most of these terms focus on the business side, indicating less emphasis on the environmental sustainability aspect. In light of the results of our bibliometric analysis, business orientation and environmental sustainability research can be viewed as a multilayered ecosystem with associated points of view on titles. The relationship between Entrepreneurial Orientation and Environmental Sustainability is crucial for modern businesses. Companies that effectively integrate EO with ES principles are more likely to thrive while contributing positively to society and the environment.

## 6 Limitations and future work

Although bibliometric analysis is an effective literature review method, it has some limitations. First, bibliometric data from the Scopus and Mendeley databases are not created exclusively for the analysis of entrepreneurial orientation with environmental sustainability. Integrating entrepreneurial orientation with environmental sustainability in firms may not be fully understood or appreciated. The results of this study provide a basis for future studies to determine the attribution of researchers to countries, institutions, collaborative publications, and disciplines. Future work in this area could focus on addressing these limitations and advancing the understanding and integration of entrepreneurial orientation and environmental sustainability by developing a framework that promotes collaboration, advocacy, and policy support. We recommend that future work include detailed business models for additional scientific mapping of the nexus between entrepreneurial orientation and environmental sustainability.
