# Supplementary material for: Scientific mapping of the nexus between entrepreneurial orientation and environmental sustainability: bibliometric analysis
Source: Front Sociol. 2025 Jan 10;9:1461840. doi: 10.3389/fsoc.2024.1461840 (PMC11758184; doi:10.3389/fsoc.2024.1461840)
Supplement: Supplementary file 1 [file Data_Sheet_1.pdf]

Appendix 1**.** Number of articles and journal types of entrepreneurial orientation and environmental sustainability from 2012 to 2022

	Symbol	Full Name of Journals	Number articles
1	Sust	Sustainability	20
2	Bus. Str. Env	Business strategy and the environment	15
3	J. Cl. Pr	Journal of Cleaner Production	11
4	Int. J. Bus. Soc. Sci	International Journal of Business and Social Science	5
5	J. Sm. B. Mag	Journal of Small Business Management	4
6	J. Bus. Ethi	Journal of Business Ethics	3
7	Entr. Res. J	Entrepreneurship research journal	3
8	Cog. Bus. Man	Cognitive Business and Management	3
9	Sup.Ch. Man	Supply Chain Management	2
10	Soc. Beh. Sci	Social and Behavioral Science	2
11	J. Sm. Bus. Entrep	Journal of Small Business and Enterprise Development	2
12	Int. J. Entrep. V	International Journal of Entrepreneurial Venturing	2
13	Int. J. Entrep. Be. Res	International Journal of Entrepreneurial Behavior & Research	2
14	Int. J. Entrep. Behav	International Journal of Entrepreneurial Behavior	2
15	Int. J. En. Econ. P	International Journal of Energy Economics and Policy	2
16	Int. J. Energy Econ	International Journal of Energy and Economics	2
17	Env. Sci. Poll. R	Environmental Science and Pollution Research	2
18	Tour. Manag	Tourism Management	1
19	Tech. For. Soc. Ch.	Technological Forecasting & Social Change	1
20	Sust. Pract	Sustainability practices	1
21	Sm.Bus. Econ	Small Business Economics	1
22	Res. Con. R.	Resource Conservation and Recycle	1
23	Organ. Sci	ORGANIZATION SCIENCE	1
24	Mat. Sci. Eng	Material Science and Engineering	1
25	Man. Res. Rev	Management Research and Resolution	1
26	**J.** Bus,Res	Journal of Business Research	1
27	J. Sustain. Tour	Journal of Sustainable Tourism	1
28	J. Environ. Sci. Sustain	Journal of Environmental Science and Sustainable Development	1
29	J. Enterprising Cult	Journal of Enterprising Culture	1
30	J. Eng. Des. Technol	Journal of Engineering, Design and Technology	1
31	J. Econ. Manag. Sci	Journal of Economics and Management Science	1
32	J. China Tour.	Journal of China Tourism	1
33	J. Bus. Ventur	Journal of Business Venturing	1
34	Int. Small Bus. J	International Small Business Journal	1
35	Int. J. Sustain. Eng	International Journal of sustainable and Engineering	1

36	Int. J. Recent Technol. Eng	International Journal of Recent Technology and Engineering	1
37	Int. J. Proj. Manag	International Journal of Project Management	1
38	Int. J. Prod. Econ	International Journal of Production Economics	1
39	Int. J. Innov. Res. Adv	International Journal of innovative and research advances	1
40	Int. J. Entrep. Small Bus	Int. J. Entrepreneurship and Small Business	1
41	Eur. J. Bus. Manag	European Journal of Business and Management	1
42	Environ. Eng. Manag. J	Environmental Engineering and Management Journal	1
43	Entrep. Sustain.	Entrepreneurship and sustainability issues	1
44	Entrep. Orit. small firm Perf	Entrepreneur Orientation and small firm performance	1
45	Creat. Educ	Creative Education	1
46	Corp. Soc. Respo. Environ. Man.	Corp Social Responsible Environment and Management	1
47	Corp. Gov	Corp of Governance	1
48	Corp Soc Respo Env. Man	Corp Social Responsible Environ Management	1
49	Clean Technol. Environ	Clean Technology and Environment	1
50	Bus. Stra. Dev	Business Strategy and Development	1
51	Asian J Bus Ethics	Asian J Bus Ethics	1
52	Am. Rev. Public Adm	American Review of Public Administration	1
53	Int. J. Environ	International Journal of Environment	1

Appendix 2. A co-occurrence network of the most keywords frequently used (Figure5)

S.no	Keywords	Occurrences	Total link strength	Clusters
1	Case Study	2	10	Cluster 1
2	Case Study, decoupling, institutional theory	2	10	
3	Decoupling	2	10	
4	Ethical Practice	2	10	
5	Institutional theory	4	12	
6	Social Sustainable	2	10	
7	Energy efficiency	2	3	Cluster 2
8	Entrepreneurial Orientation	6	9	
9	Ghana	2	3	
10	Malaysia	2	3	
11	Sustainable Orientation	3	1	
12	Sustainable Development	4	4	Cluster 3
13	Green Entrepreneurial Orientation	5	16	
14	Green Supply Chain Management	2	10	
15	Market Orientation	2	10	
16	Operational Management	2	10	
17	Sustainable Performance	3	14	
18	Textile Industry	2	10	
19	Sustainability	6	4	Cluster 4
20	Follows	2	6	
21	Green Entrepreneurship	2	3	
22	Customer Orientation	2	2	Cluster 5
23	Sustainable Entrepreneurial Orientation	2	2	
24	Reference in this paper	2	6	
25	Should be made as	2	6	

Note: the minimum number of occurrence of terms 2; of the 183 terms, 25 meet the thresholds, and 25 numbers of terms were obtained from full counting analysis. Summary of co-occurrences network were 25 items, 5 clusters, 54 links, and 91 total link strength

Appendix 3. Overlay visualization occurrences of terms in abstract fields based searching of the total number of articles (Fig. 6)

S.no	Abstract field	Occurrences	Relevance	Clusters
1	Business	18	0.59	Cluster 1
2	Entrepreneurial activity	10	2.35	
3	Entrepreneurship	26	0.75	
4	Paper	15	1.18	
5	SMES	13	0.71	
6	Sustainable Development	14	1.24	
7	Entrepreneur	19	0.63	
8	Entrepreneurial Orientation	32	0.82	Cluster 2
9	Natural Resources	11	1.03	
10	Performance	33	0.76	
11	Relationship	29	0.83	
12	Resource	11	0.99	
13	View	15	1.13	

Summary: 13 items, 2 clusters, 73 Links, and 437 total link strength

Appendix 4. Network visualization occurrences of terms in title and abstract fields based searching of the total number of articles (Fig. 7).

S.no	Title and abstract fields	Occurrences	Relevance	Clusters
1	Entrepreneurship	27	1.18	Cluster 1
2	Entrepreneur	19	0.44	
3	Implication	21	0.75	
4	Opportunity	19	0.65	
5	Paper	16	1.27	
6	Sustainability	40	0.34	
7	Sustainable Entrepreneurship	11	0.64	
8	Entrepreneurial Orientation	47	0.78	Cluster 2
9	Environment	21	0.65	
10	Environmental Performance	13	1.50	
11	Natural Resources	11	1.67	
12	Performance	42	0.82	
13	View	15	1.29	
14	Entrepreneurial activity	10	1.54	Cluster 3
15	Medium enterprise	10	1.50	
16	Medium size enterprise	10	1.19	
17	Resource	15	0.38	
18	SMES	16	1.48	
19	Sustainable Development	21	0.93	

Summary: 19 items, 3 clusters, 160 links, and 805 total link strength

Appendix 5. Network visualization occurrences of terms in title field based searching of the total number of articles (Fig.8)

S.no	Title field	Occurrences	Relevance	Clusters
1	Green Entrepreneurial Orientation	7	0.24	Cluster 1
2	Green Supply chain Management	5	0.29	
3	Impact	12	0.26	
4	Environment	6	0.33	Clusters 2
5	Environmental Orientation	5	0.36	
6	Role	14	0.33	Cluster 3
7	Sustainable Entrepreneurship	5	0.36	
8	Mediator	5	1.94	
9	SMEs	9	2.47	
10	Malaysia	5	1.94	

Note: 10 items, 3 clusters, 12 links, and 20 total link strength
